# Multiple somatic symptoms in primary care patients: a cross-sectional study of consultation content, clinical management strategy and burden of encounter

**DOI:** 10.1186/s12875-016-0478-z

**Published:** 2016-07-30

**Authors:** Mette T. Rask, Anders H. Carlsen, Anna Budtz-Lilly, Marianne Rosendal

**Affiliations:** Research Unit for General Practice, Department of Public Health, Aarhus University, Bartholins Allé 2, 8000 Aarhus C, Denmark

**Keywords:** General practice, Somatoform disorders, Medically unexplained symptoms, Signs and symptoms, Referral and consultation, Difficult patient encounters

## Abstract

**Background:**

Consultations involving patients with multiple somatic symptoms may be considered as challenging and time-consuming by general practitioners (GPs). Yet, little is known about the possible links between consultation characteristics and GP-experienced burden of encounter. We aimed to explore consultation content, clinical management strategies, time consumption and GP-experienced burden of encounters with patients suffering from multiple somatic symptoms as defined by the concept of bodily distress syndrome (BDS).

**Methods:**

Cross-sectional study of patient encounters in primary care from December 2008 to December 2009; 387 GPs participated (response rate: 44.4 %). Data were based on a one-page registration form completed by the GP and a patient questionnaire including the 25-item BDS checklist for somatic symptoms. Using logistic regression analyses, we compared patients who met the BDS criteria with patients who did not.

**Results:**

A total of 1505 patients were included (response rate: 55.6 %). Health problems were less frequently reported as ‘new’ in patients with BDS (odds ratio (OR) = 0.73, 95 % confidence interval (CI): 0.54; 0.97). Medical prescriptions and referral rates were comparable in the two patient groups. Consultations focusing on mainly biomedical aspects were less frequent among patients with BDS (OR = 0.31, 95 % CI: 0.22; 0.43), whereas additional biomedical and psychosocial problems were more often discussed. GPs were more likely to ensure continuity of care in BDS patients by watchful waiting strategies (OR = 2.32, 95 % CI: 1.53; 3.52) or scheduled follow-up visits (OR = 1.61, 95 % CI: 1.09; 2.37). Patients with BDS were found to be more time-consuming (OR = 1.77, 95 % CI: 1.26; 2.48) and burdensome (OR = 2.54, 95 % CI: 1.81; 3.55) than patients without BDS. However, after adjustments for biomedical and psychosocial content of the consultation, the identified differences for time consumption and burden were no longer statistically significant.

**Conclusions:**

Patients with BDS represent higher care complexity in terms of biomedical and psychosocial needs. GPs seem to allow space and time for discussing these issues and to aim at ensuring continuity in care through watchful waiting or scheduled follow-up consultations. However, the reported GP-experienced burden call for professional development.

## Background

Somatic symptoms that are not attributable to any conventionally defined disease are highly prevalent in general practice [1, 2]. These represent a spectrum ranging from self-limiting symptoms to more severe and persistent conditions, which are often characterized by multiple symptoms. Multiple somatic symptoms challenge both patients and health-care professionals as such conditions often remain ‘unexplained’ after extensive investigations. Furthermore, the symptoms can be debilitating and difficult to treat or may occur in combination with anxiety and depression [3–8].

A biopsychosocial assessment is recommended for early recognition of conditions of multiple somatic symptoms [9]. However, general practitioners (GPs) tend to focus on the biomedical aspects in the primary evaluation of multiple somatic symptoms, while psychological issues are more often addressed in follow-up consultations [10, 11]. Previous studies suggest that many GPs find it troublesome and time-consuming to deal with multiple somatic symptoms, but most previous studies on GP experience have been qualitative [10, 12, 13], have explored the GPs’ general attitudes to patients with medically unexplained symptoms [14, 15] or have addressed patients with mental health problems [16], difficult encounters [17] or long consultations [18]. Two studies have found that the GP-rated complexity of the clinical encounter tends to increase with number of somatoform symptoms [19, 20]. Nevertheless, it remains poorly investigated how conditions of multiple somatic symptoms are associated with consultation content, clinical management strategy, time consumption and the GP’s experience of the encounter.

Numerous terms and diagnoses have been applied to multiple somatic symptoms without a biomedical explanation, e.g., medically unexplained symptoms, somatisation disorder, somatoform disorder and functional somatic syndromes such as fibromyalgia, chronic fatigue and irritable bowel syndrome [21]. A new concept, bodily distress syndrome (BDS), was recently introduced. BDS derives from empirical studies and has been shown to capture the vast majority of patients with somatoform disorders and functional somatic syndromes [22, 23]. This new unifying concept has been incorporated into the current draft of the International Classification of Diseases for Primary Health Care 11^th^ revision (ICD-11-PHC) and has been found acceptable to clinicians in the first field trials [24].

Our study addressed patients with BDS in primary care, and the aim of the study was two-fold. First, we aimed to describe the consultation content and the GP management of patients with BDS. Second, we wanted to explore the time consumption and the GP-rated burden of these patients. The study was part of a large cohort study of primary care encounters.

## Methods

### Design and setting

We conducted a cross-sectional study based on a Danish questionnaire survey on reasons for encounter and disease patterns in general practice; all GPs in the Central Denmark Region were invited to participate in this survey [25]. The Central Denmark Region is a mixed rural and metropolitan area with nearly 1.3 million inhabitants served by approximately 870 GPs. The Danish healthcare system is almost entirely tax-funded, and about 98 % of all Danish residents are listed with a general practice.

### Sample

A total of 387 GPs (response rate: 44.4 %) participated in the questionnaire survey, which was conducted from December 2008 to December 2009. Participants were comparable to non-participants in terms of type of clinic (solo or partnership practices) and age and gender of listed patients. However, participants were more likely to be men and to have less than 20 years of practice experience [25]. The participating GPs completed a one-page registration form for each of the consulting patients on one randomly assigned day (or two half-day sessions). Randomisation of days was performed by the research group. The GPs received a remuneration of EUR 32 for participation and an additional EUR 3 for each completed registration form.

**Fig. 1:**
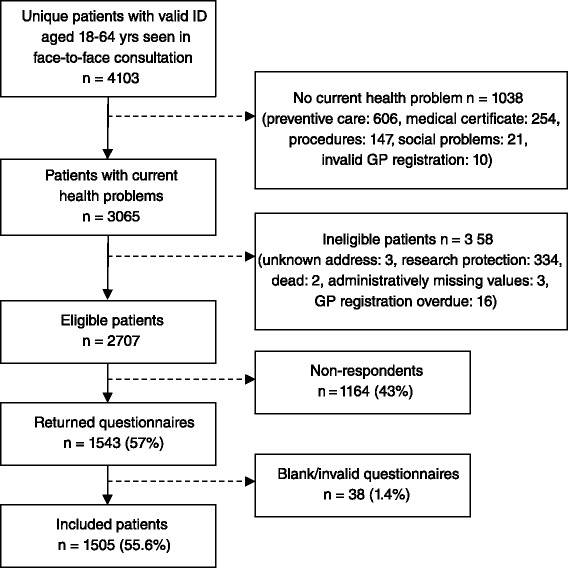
Flow chart illustrating the patient inclusion

The GPs registered the patient’s civil registration number (ID) on the registration form. After the consultation, unique patients with a valid ID received a postal questionnaire inquiring about bodily distress symptoms and self-evaluated health. For the purpose of the present study, we included only patients aged 18–64 years who had visited their GP due to a current health problem and who had completed the patient questionnaire (see Fig. 1).

### Outcome measures

The GPs stated reason for encounter and diagnosis (final conclusion about the health problem) in the registration form at the end of the consultation. All diagnostic information was subsequently coded by the research group using the International Classification of Primary Care (ICPC) [26]. We dichotomised both ‘reason for encounter’ and ‘diagnosis’ at the end of the consultation according to either ‘specific disease/disorder’ (i.e., diagnostic criteria for specific disease/disorder fulfilled) or ‘symptom’ (i.e., symptom cannot be ascribed a specific disease or disorder) in line with ICPC criteria.

Information on whether the main health problem was new (yes/no) was obtained from the GP registration form. Each participating GP indicated chosen management of the health problem in terms of prescription of medication (yes/no), diagnostic imaging (yes/no) and referral to specialized care (yes/no). Furthermore, the GP was asked to state how the consultation had been closed: 1) completed without further action, 2) scheduled follow-up or 3) watchful waiting.

The complexity of delivered care was explored by two questions. First, the GP was asked to assess the importance of biomedical, psychological and social aspects in the consultation on a rating scale summing to 100 %. Second, the GP stated the number of biomedical, psychological and social problems addressed in addition to the main problem. The content of each consultation was grouped into primarily biomedical or not (yes/no) for the analyses. Additional problems were grouped into biomedical problems (yes/no) or psychosocial problems (yes/no).

The GP was asked to state the duration (in minutes) of the consultation. Duration was subsequently categorized into ≤ 15 min, 16–30 min and ≥ 31 min. Finally, the GP assessed the burden of the consultation on a scale ranging from 0 to 10, and three groups were created for the analyses by using the 20 and 80 % percentile as cut-offs: low (1–2), medium (3–6) and high (7–10) burden.

### The BDS checklist

The patient questionnaire comprised the BDS Checklist, which explores the presence of 25 symptoms from four main symptom groups: cardiopulmonary, gastrointestinal, musculoskeletal and general symptoms [27]. The patient was asked to rate on a 5-point Likert-type scale how much s/he had been bothered by each of the 25 listed symptoms within the past four weeks. Response categories were: ‘not at all’, ‘a bit’, ‘somewhat’, ‘quite a bit’ and ‘a lot’. A validation of the BDS checklist has shown that four symptoms from at least one of the four BDS symptom groups indicate probable BDS [27]. In the present study, patients were considered to suffer from clinically significant BDS if they had been bothered at least ‘somewhat’ by at least four symptoms, and they reported ‘fair’ or ‘poor’ health as measured by a single general-health item from the 12-Item Short-Form Health Survey (SF-12) [28]. BDS status was recorded only for patients who completed the SF-12 item and at least 50 % of the BDS checklist items.

### Statistical analyses

Descriptive statistics were performed by using Student’s t-test for data following a normal distribution, Mann–Whitney U-test for non-normally distributed data and chi-square test for categorical data.

Potential associations between BDS and the outcomes of interest were investigated by three logistic regression models with BDS as the independent variable; Model 1: univariate analyses of all outcomes of interest, Model 2: multivariate analyses of all outcomes of interest (adjusted for patient age, gender, education and chronic disorders grouped as none, somatic only, somatic and psychiatric, or psychiatric disorders only) and Model 3: multivariate analyses of time consumption and burden (adjusted for the above-mentioned patient characteristics, whether the health problem was new or already known, and the biomedical and psychosocial content of the consultation). Information on covariates was obtained from the GP registration form, except for information on education which was retrieved from Statistics Denmark [29].

The results of the logistic regression analyses were presented as odds ratios (OR) with 95 % confidence interval (CI) using robust variance estimates to account for clustering at GP level. Statistical significance was set at *p* < 0.05. Statistical analyses were conducted by Stata, version 13.1 (StataCorp LP, College Station, TX, USA).

## Results

A total of 1505 patients (response rate: 55.6 %) completed the questionnaire and were included in the study (Fig. 1). Non-respondents were statistically significantly more likely to be younger (*p* < 0.001), male (*p* = 0.022), living alone (*p* < 0.001) and to have fewer years of education (*p* < 0.001). No differences were found for chronic disorders (*p* = 0.680).

Among the included patients, the prevalence of BDS was 17.5 % (*n* = 264). Patients with BDS reported more somatic symptoms than patients without BDS (median = 11 vs. 2, *p* < 0.001), and they were more likely to be older, to be female and to have fewer years of education (Table 1). The group of patients with BDS comprised more individuals with chronic disorders than the group without BDS (63.3 % vs. 36.8 %, *p* < 0.001). In the BDS group, 20.5 % suffered from a chronic psychiatric disorder, while this was the case for only 8.6 % in the non-BDS group (Table 1).

**Table 1:** Patient sociodemographics and clinical characteristics according to BDS status

	Total (*n* = 1505)		No BDS (*n* = 1241)		BDS (*n* = 264)		*p*
Bodily distress symptoms, median, IQR	3	1;7	2	0;5	11	7;14	<0.001
Age; mean, SD	45.6	12.9	45.1	13.2	47.7	11.1	0.001
	n	%	n	%	n	%	
Gender							
Male	535	35.5	459	37.0	76	28.8	
Female	970	64.5	782	63.0	188	71.2	0.012
Marital status							
Married/cohabitating	1171	77.8	969	78.1	202	76.5	
Living alone	334	22.2	272	21.9	62	23.5	0.578
Education							
≤ 10 y	377	25.3	276	22.5	101	38.9	
> 10y & ≤15 y	784	52.7	664	54.0	120	46.2	
> 15y	328	22.0	286	23.5	39	15.0	<0.001
Chronic illness							
None	881	58.5	784	63.2	97	36.7	
Somatic only	463	30.8	350	28.2	113	42.8	
Somatic and psychiatric	55	3.7	35	2.8	20	7.6	
Psychiatric only	106	7.0	72	5.8	34	12.9	<0.001

*BDS* bodily distress syndrome, *IQR* interquartile range, *SD* standard deviation

Missings: Education *n* = 16

### Consultation characteristics and management strategy

Patients with BDS less frequently presented a new health problem (OR = 0.73, 95 % CI: 0.54; 0.97) compared to patients without BDS. The two patient groups had comparable rates of symptoms presented as reason for encounter and were equally likely to be labelled by a symptom diagnosis at the end of the consultation (i.e., the symptom could not be ascribed to a specific disease or disorder) (Table 2).

**Table 2:** Consultation content and GP management, time consumption and experienced burden according to BDS status

	Total (*n* = 1505)		No BDS (*n* = 1241)		BDS (*n* = 264)					
	n	%	n	%	n	%	OR _Crude_	(95 % CI)	OR _Adjusted_	(95 % CI)
Symptom as reason for encounter	907	64.7	750	64.4	157	66.2	1.08	(0.79;1.48)	1.33	(0.95;1.86)
New health problem	810	56.6	696	59.0	114	45.2	0.57	(0.44;0.75)	0.73	(0.54;0.97)
Symptom as end diagnosis	552	36.7	454	36.6	98	37.1	1.02	(0.78;1.34)	1.16	(0.87;1.55)
Prescription of medication	541	36.0	442	35.6	99	37.5	1.08	(0.81;1.46)	1.00	(0.74;1.36)
Referral to medical specialist (inpatient or outpatient)	203	13.5	159	12.8	44	16.7	1.36	(0.95;1.96)	1.35	(0.93;1.96)
Referral to diagnostic imaging	79	5.2	68	5.5	11	4.2	0.75	(0.40;1.39)	0.81	(0.42;1.54)
Consultation closure										
Encounter completed	363	25.8	324	28.0	39	15.8	Reference		Reference	
Scheduled follow-up	708	50.4	572	49.4	136	55.1	1.98	(1.38;2.84)^a^	1.61	(1.09;2.37)^a^
Watchful waiting	335	23.8	263	22.7	72	29.2	2.27	(1.51;3.42)^b^	2.32	(1.53;3.52)^b^
Mainly biomedical issues	1096	75.4	963	80.3	133	52.6	0.27	(0.21;0.36)	0.31	(0.22;0.43)
Additional biomedical problems	458	30.4	357	28.8	101	38.3	1.53	(1.14;2.07)	1.38	(1.01;1.90)
Additional psychosocial problems	218	14.5	147	11.9	71	26.9	2.74	(1.93;3.89)	2.08	(1.40;3.09)
Consultation duration										
≤ 15 min	1190	80.4	1011	82.7	179	69.4				
16–30 min	279	18.8	203	16.6	76	29.5				
31+ minutes	12	0.8	9	0.7	3	1.2	2.10	(1.54;2.87)^c^	1.77	(1.26;2.48)
GP-experienced burden										
Low	485	32.9	440	36.5	41	15.9				
Middle	754	51.2	617	50.8	137	53.1				
High	234	15.9	154	12.7	80	31.0	3.10	(2.24;4.29)^d^	2.54	(1.81;3.55)

*BDS* bodily distress syndrome

Missings: Reason for encounter *n* = 104, New health problem *n* = 74, Consultation closure *n* = 99, Mainly biomedical issues *n* = 52, Consultation duration *n* = 24, Burden *n* = 32

OR adjusted: Adjusted for patient age, gender, education and chronic illness

^a,b^Reference group: Encounter completed; Multinomial regression analysis

^c^Test of ≤15 min vs. 16+ min due to low cell count

^d^Test of high vs low/middle

GPs prescribed medication in more than one third of all consultations, and they referred 5.2 % of the patients to diagnostic imaging and 13.5 % to a medical specialist (inpatient or outpatient). The GPs did not appear to manage patients with BDS differently from patients without BDS in terms of prescriptions and referrals (Table 2). Yet, the GPs more often chose to end consultations with BDS patients with strategies of watchful waiting (OR = 2.32, 95 % CI: 1.53; 3.52) or scheduled follow-up visits (OR = 1.61, 95 % CI: 1.09; 2.37), whereas consultations with patients without BDS were more often completed without further action.

Consultations with BDS patients were also more likely to address both biomedical and psychosocial issues, and the GPs rated fewer consultations with BDS patients as mainly biomedically oriented (52.6 % vs. 80.3 %, OR = 0.31, 95 % CI: 0.22; 0.43). Patients with BDS presented more additional problems than patients without BDS, both biomedical problems (38.3 % vs. 28.8 %, OR = 1.38, 95 % CI: 1.01; 1.90) and psychosocial problems (26.9 % vs. 11.9 %, OR = 2.08, 95 % CI: 1.40; 3.09).

### Time consumption and burden

Patients with BDS were more likely to have long consultations (OR = 1.77, 95 % CI: 1.26; 2.48) and to represent high burden (OR = 2.54, 95 % CI: 1.81; 3.55) than patients without BDS, even after adjustment for chronic illness (Table 2). After further adjustment for consultation characteristics, we could no longer detect a statistically significant relationship between BDS and time consumption or burden (Table 3). The multivariate analyses showed that consultations of mainly biomedical orientation were more likely to be characterised by short duration and low burden, while consultations in which additional problems were addressed (biomedical or psychosocial) were more likely to be characterised by long duration and high burden. Chronic somatic or psychiatric disorders in themselves did not seem to explain the findings on consultation duration and perceived burden. Consultation duration was found to be strongly related to the GP-experienced burden; patients with a long consultation were four times more likely to represent a high burden (Table 3).

**Table 3:** Multivariate analyses of long consultations (*n* = 1354) and GP-perceived high burden (*n* = 1330)

	Consultation duration 16+ min		GP-experienced high burden	
	OR	(95 % CI)	OR	(95 % CI)
Bodily distress syndrome	1.06	(0.72;1.57)	1.53	(0.99;2.37)
New health problem	0.97	(0.69;1.37)	1.19	(0.84;1.69)
Mainly biomedical issues	0.20	(0.14;0.28)	0.37	(0.25;0.57)
Additional biomedical problems	2.03	(1.45;2.85)	1.47	(1.00;2.15)
Additional psychosocial problems	2.25	(1.49;3.39)	2.00	(1.35;2.99)
Consultation duration >15 min			4.47	(2.99;6.70)
Age	1.00	(0.99;1.02)	1.00	(0.99;1.01)
Female gender	1.10	(0.79;1.53)	1.11	(0.75;1.64)
Education >10 years	1.18	(0.84;1.66)	0.73	(0.48;1.12)
Chronic illness				
Somatic only	1.26	(0.90;1.78)	1.26	(0.86;1.83)
Somatic and psychiatric	1.01	(0.46;2.32)	2.31	(0.95;5.66)
Psychiatric only	1.50	(0.85;2.61)	1.19	(0.65;2.18)

## Discussion

### Summary

In this study, we explored consultation content, clinical management strategy, time consumption and GP-experienced burden related to patients with BDS. Consultations with BDS patients more often addressed additional and multifaceted problems, and these patients were found to be more time-consuming and burdensome than patients without BDS. Yet, additional problems or psychosocial issues addressed in the consultation seemed to explain these differences.

### Strengths and limitations

One of the strengths of this study was that we included data for a large number of encounters between GPs and patients. We obtained otherwise unavailable information about consultation characteristics, clinical management strategy and GP experience of encounter. Furthermore, the GP registration forms were completed immediately after each consultation, which reduced the risk of recall bias. The study does, however, also have several limitations.

First, the response rate is of concern. Our sample comprised more females, and they were older, more often married or cohabitating and better educated than non-respondents. It is not clear how this might have impacted the associations between the studied variables. On the one hand, the prevalence of BDS may have been overestimated as patients with BDS were older and more often female, and, on the other hand, the prevalence may have been underestimated as patients with BDS generally had shorter education.

Second, we did not make a clinical diagnosis of BDS, and patients may thus have been misclassified if their multiple somatic symptoms were due to a specific disease or disorder. The BDS Checklist has been shown to indicate probable BDS. When adding a further criterion on impairment, the BDS Checklist also appears to capture a group of patients with poor physical and mental health and high use of medical services [27, 30]. Patients with BDS were no more likely to be labelled with a diagnosis for a specific disease or disorder at the end of the consultation than patients without BDS, and adjustment for chronic disorders did not influence our findings. Thus, misclassification is unlikely to have affected the study conclusions.

Third, included patients completed the BDS Checklist after the consultation. Therefore, the GP did not know the patient’s BDS status during the consultation and has not been influenced by such knowledge. However, this also means that we do not know whether the reason for encounter, the main problem addressed or any of the additional problems presented in the consultation were related to BDS. As a consequence, our findings do not reflect how GPs manage BDS, but rather how they manage patients with probable BDS presenting a health problem. Future research is needed on BDS as reason for encounter and GP management of BDS.

### Comparison with existing literature

Although previous studies have shown that patients with multiple somatic symptoms display high health-care use [3, 31, 32], we were not able to detect any differences in referral rates between patients with and without BDS, at least not on the basis of only one encounter. However, the GPs more often applied a watchful waiting strategy or scheduled a follow-up visit for patients with BDS, which may have contributed to higher health-care use in primary care at a later point in time. Terms like ‘high users’ or ‘frequent attenders’ tend to have negative connotations. Nevertheless, the GP’s chosen strategy of watchful waiting or follow-up visit may actually be preferable. First, it may enhance the continuity of care, which is recommended in the clinical guidelines for patients with multiple symptoms [9, 33, 34]. Second, management within rather than outside the practice may reduce the risk of unnecessary investigations and treatments, which may enhance patient safety and yet ensure that management costs are kept at low level [9, 35].

Previous studies have shown that psychiatric disorders, multiple symptoms and functional impairment are more prevalent among primary-care patients who are rated as difficult by their GP [17, 19, 20] and that consultations introducing mental health problems are more time-consuming [16]. In line with these findings, our results showed that consultations with patients with BDS were more often of long duration and high burden. This seemed to be explained by more psychosocial-oriented consultations concerning additional problems. Our findings may indicate that GPs allow time for addressing additional problems and psychosocial issues. A review has indicated that increased consultation length may lead to more accurate assessment of psychological problems [36]. Yet, based on our study, we cannot say whether the longer consultations were related to better quality of care.

The majority of patients with BDS came due to health problems which had earlier been recognised by their GP. Our results indicate that most GPs take on the responsibility as case manager for these patients although some may find it burdensome. Personal continuity may support symptom assessment and management decisions, especially in patients with complex and psychological problems. However, when a patient does not get better, and the GP feels stuck, the personal relationship may be experienced as a burden [37]. Several factors may contribute to the GP-experienced burden for patients with BDS; the GP may often feel unable to provide adequate explanations for multiple somatic symptoms, may feel that s/he lacks sufficient management strategies to handle patients with multifaceted problems and may feel pressured by time restrictions [10, 12, 13]. We found a strong association between consultation duration and experienced burden.

## Conclusions

Patients with BDS represent higher care complexity, both in regard to biomedical and psychosocial needs. GPs seem to allow time for discussing these needs and ensuring continuity in care through watchful waiting strategies and scheduled follow-up consultations. However, the identified GP-experienced burden suggests a need for continuing professional development to ensure that both patients with multiple somatic symptoms and the psychosocial aspects of care are embraced and that GPs are better equipped to fill the case manager role.

## Abbreviations

BDS, bodily distress syndrome; CI, confidence interval; EUR, euro; GP, general practitioner; ICD-11-PHC, International Classification of Diseases for Primary Health Care 11^th^ revision; ICPC, International Classification of Primary Care; ID, civil registration number; IQR, interquartile range; OR, odds ratio; SD, standard deviation; SF-12, 12-Item Short-Form Health Survey
